# Minimally invasive selective caries removal: a clinical guide

**DOI:** 10.1038/s41415-023-5515-4

**Published:** 2023-02-24

**Authors:** Zi En Lim, Henry F. Duncan, Advan Moorthy, David McReynolds

**Affiliations:** 41415157234001 General Dental Practitioner, Dublin, Ireland; 41415157234002 Professor/Consultant in Endodontics, Trinity College Dublin, Ireland; 41415157234003 Prosthodontist, Private Practice, Ireland; 41415157234004 Academic Prosthodontist and Assistant Professor in Restorative Dentistry, Dublin Dental University Hospital, Ireland

## Abstract

An evolving understanding of the carious process, along with new research in adhesive restorative materials, has led to a more conservative, minimally invasive and biologically-based approach to managing dental carious lesions. The growing volume of literature has also demonstrated prognostic success in the selective caries excavation technique, subsequently preventing excessive tooth structure removal and injury to the dentine-pulp complex, which maintains pulp vitality and improves the long-term prognosis of the tooth. However, at present, there remains a limited volume of high-quality evidence to support selective caries removal, which subsequently could partly explain some resistance to its use in clinical practice. This clinical technique guide aims to demonstrate the management of carious lesions of moderate-to-deep depth in permanent teeth based on current minimally invasive dental literature.

## Key points

- The non-selective caries excavation technique, involving complete removal of caries-affected dentine, is considered overtreatment, being needlessly invasive and outdated.
- Selective caries excavation disrupts the carious biofilm ecology while simultaneously minimising over-preparation of tooth structure and injury to the dentine-pulp complex.
- Development of the peripheral seal zone is critical in order to create a hermetic seal when methacrylate resin-based composites are used to restore teeth.
- Bonding protocols are very technique-sensitive and necessitate a meticulous approach to their execution to ensure optimal bond strengths are achieved.

## Introduction

In contemporary dental practice, a clearer understanding of the carious process and an increased volume of clinical evidence in operative dentistry has informed the profession's approach to the management of carious lesions. The traditional G. V. Black's cavity designs from the twentieth century adopted an 'extension for prevention' approach that involved the surgical removal of both demineralised carious infected dentine and any tooth structure which had been affected by the carious process.^1^ However, this cavity design was traditionally intended for dental amalgam restorations, where further tooth structure removal is required to create cavity retention and resistance form. We now know that the removal of so-called caries-affected dentine is not mandated in the treatment of the carious lesion, while developments in adhesive bioactive/bio-interactive restorative materials and an awareness of the remineralisation potential of dentine has led to a decreased reliance in Black's cavity designs to directly restore teeth.^2^^,^^3^ Furthermore, the Minamata Treaty has advised the phasing-down of amalgam restorations due to environmental concerns over mercury levels.^4^ Thus, a paradigm shift based on a research-led approach has resulted in the adoption of minimally invasive dentistry in managing carious lesions. Despite these advances in our understanding, there remains confusion, debate and resistance to change when translating these ideas into clinical practice. This is partly caused by the lack of high-quality, definitive scientific evidence behind this technique, particularly in the permanent dentition, since most studies are obtained from trials on the primary dentition.^5^ For this reason, the SCRiPT trial is currently being undertaken to clarify confusion around this subject.^5^ Using the currently available literature pertaining to selective caries removal, this clinical technique guide aims to visually document the procedural steps and to justify its rationale when restoring moderate-to-deep depth carious lesions with direct methacrylate resin composite restorations in clinical practice.

## The advantages of selective caries removal

Minimally invasive dentistry encompasses conservative operative techniques that preserve hard and soft tissues when managing cavitated carious lesions.^6^ Decades ago, non-selective caries removal had been the recommended treatment modality, which encompasses removal of all carious tooth structure to sound enamel and dentine. However, carious lesions can and should be managed conservatively first and foremost by controlling those aetiological factors of the carious process. Such strategies include diet modification, biofilm disruption and hermetically sealing cariogenic biofilm from its nutrient supply.^7^^,^^8^^,^^9^ Therefore, from an operative perspective, selectively excavating carious tissue can be effective without having to completely eradicate the entire bacterial population.

In the short-term, the non-selective caries excavation approach involves unnecessary over-preparation of tooth structure with resultant damage to the dentine-pulp complex.^8^ In the long-term, the unnecessary excessive removal of healthy tooth structure tends to compromise the mechanical integrity of the tooth, making it more prone to potentially catastrophic 'cracks', fractures and their associated sequalae.^10^^,^^11^

Particularly in the deeper cavity, excessive removal of tooth structure would tend to increase the risk of a pulpal exposure, resulting in irreversible damage to the odontoblastic palisade and death of primary odontoblasts.^8^^,^^9^ Selective caries removal, on the other hand, arrests carious lesion activity while simultaneously reducing the risk of pulpal exposure and preserving the odontoblastic palisade; a crucial area that induces the more ordered deposition of reactionary rather than reparative tertiary dentinogenesis.^8^^,^^9^^,^^10^ It also reduces risk of bacterial ingress into the pulp, thereby maintaining pulp vitality. This maximises the prognosis of the tooth and should reduce long-term management costs and burden associated with teeth.^2^^,^^10^^,^^11^

Although dentine bonding to so-called caries-infected or caries-affected dentine is weaker, this is thought to be clinically insignificant as the appropriately prepared cavity should be surrounded by sound enamel and dentine with which one can consequently achieve high bond strengths and a hermetic seal when methacrylate resin-based adhesives are used.^12^

## Clinical technique

**Fig. 1:**
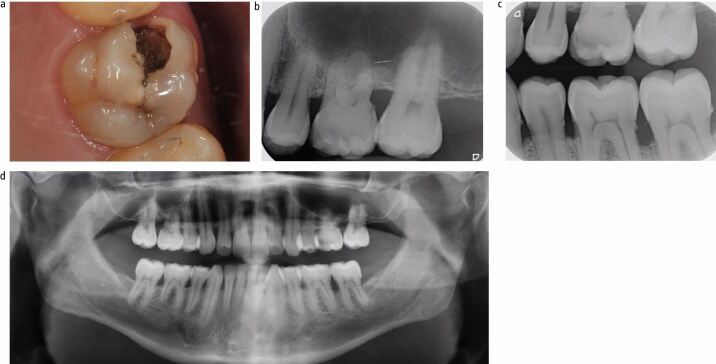
a, b, c, d) The pre-operative presentation of the 26 illustrates an ICDAS II code 06 carious lesion, with an ICDAS/ICCMS radiographic code RB4. This carious lesion can be described as a moderately deep cavitated carious lesion. This type of presentation is a very common occurrence in routine general dental practice

The patient was a 37-year-old man in good health. The patient presented with an asymptomatic cavity, which he noticed developed spontaneously when chewing food, days before presentation. The patient was most likely asymptomatic due to the dynamic reparative response of the dentine-pulp complex, thus blocking the early stage of bacterial invasion through the dentinal tubules towards the pulp. Intraoral examination revealed a partially dentate patient who was missing all first permanent pre-molars, most likely due to a history of orthodontic treatment. An extensive, distinct cavity with visible dentine was noted clinically on the mesial and occlusal surfaces of the 26 (Fig. 1a) and it did not appear to be previously restored (International Caries Detection and Assessment System [ICDAS] II code 06).^13^ The dentine appeared glossy and felt soft on gentle probing, which is suggestive of active carious progression. The tooth displayed physiological mobility, was not tender to percussion and had a non-lingering response to cold and electrical pulpal sensibility testing.

The radiographic findings of the carious lesion were consistent with the clinical diagnosis. The radiolucency attributed to the carious lesion extends to the middle third of the dentine (Figures 1b and 1c) giving it an International Caries Classification and Management System [ICCMS]/ICDAS radiographic score of RB4.^14^ Therefore, the carious lesion can be described as a moderately deep cavitated carious lesion.^2^

## Initial treatment

The patient also presented with Stage III Grade C periodontal disease which was exacerbated by root shortening (Fig. 1d). As such, the patient has concurrently undergone four quadrants of non-surgical root surface debridement in the context of interdisciplinary care with a periodontist.

Selective caries excavation would be conducted on the 26 in order to halt the carious process and prevent potential carious progression towards the pulpal tissues. A direct methacrylate resin-based composite restoration was the restorative material of choice. However, in the long-term, an assessment for cuspal coverage restoration for the 26 may be considered, providing periodontal and carious process stabilisation has been achieved.

## Selective caries removal

**Fig. 2:**
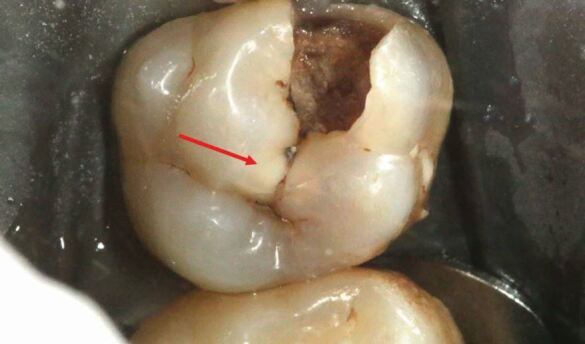
Note shadowing of underlying carious lesion occlusally around the fissure. Rubber dam isolation is an essential aspect of treatment, which becomes particularly salient when restoring teeth with moisture-sensitive restorative materials such as methacrylate resin-based composites. A strict asepsis protocol optimises treatment outcomes in vital pulp therapy, even when the pulp is not directly exposed

When placing a direct methacrylate resin-based composite restoration, moisture control and a strict asepsis protocol is essential, particularly as blood, saliva and gingival crevicular fluid will affect the adhesion of the restorative material, thereby increasing the chance of microleakage.^11^ After buccal and palatal infiltration with 2% lidocaine 1:80,000 adrenaline, rubber dam isolation was used in order to separate the operative field from the oral fluids, and to improve visual and mechanical access (Fig. 2). The 27, 26 and 25 were isolated with a W14 clamp (Ivory, Kulzer, Helsingborg, Sweden) secured on tooth 27.

The 2019 European Society of Endodontics (ESE) position statement defines two selective caries excavation endpoints: selective caries to soft dentine or to firm dentine.^15^ The decision of an appropriate caries excavation endpoint is determined by the depth of the carious lesion. In moderately deep carious lesions, selective removal of carious tissue to firm dentine is recommended.^2^ This means that the dentine situated on the pulpal wall should be leathery, while cavity margins and peripheral dentine should be caries-free and prepared to sound hard dentine.^2^ Leathery dentine is described clinically as dentine that doesn't deform when an instrument is pressed onto it and has a slight 'tackiness'.^2^^,^^12^ With hard dentine, a pushing force needs to be used to engage the dentine and a scratchy sound, known as '*cri dentinaire'*, can be heard.^2^^,^^12^^,^^16^

There are also two available selective caries excavation methods which have been recommended by the ESE: the one-stage or two-stage stepwise technique.^15^ The one-stage approach to caries excavation appears to have a more favourable long-term success compared with the two-step method.^17^ However, there is currently insufficient evidence to definitively advocate its superiority due to the lack of recalls evaluating outcomes of the stepwise technique.^15^ The inability to adequately compare success outcomes between the two techniques has therefore incited controversy within the dental profession. In this case example, the one-step caries excavation technique was employed, thereby avoiding the need of a later appointment for re-entry into the tooth and subsequent risk of further iatrogenic tooth structure loss upon removal of a temporary restoration, which would have otherwise been necessitated with the step-wise technique.

**Fig. 3:**
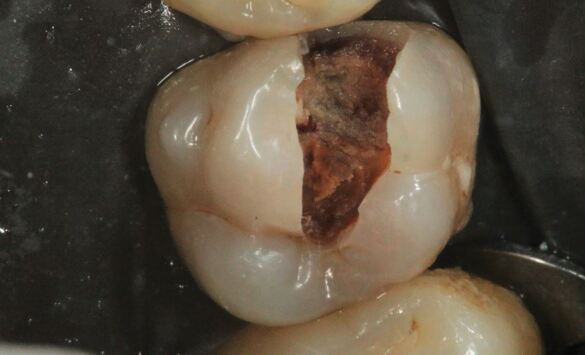
Access form is developed through the removal of carious enamel using a diamond fissure bur in a turbine handpiece under copious water coolant

**Fig. 4:**
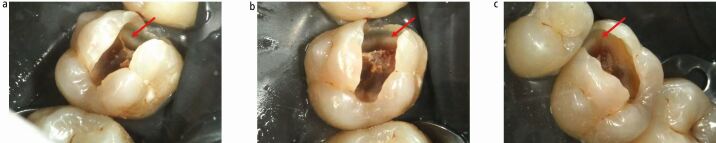
a, b, c) Initial clearing of the amelodentinal junction using the largest steel rosehead bur that can fit within the carious cavity. The preparation is carried out using a conventional handpiece under copious water coolant. Note that at this stage, the carious tooth structure is cleared from its periphery whilst the pulpal wall remains unprepared

**Fig. 5:**
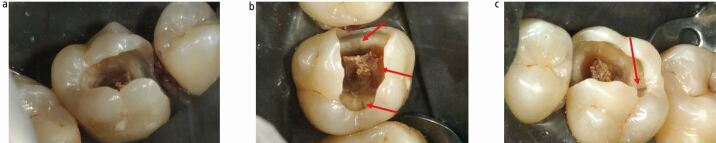
a, b, c) Definitive clearing of the amelodentinal junction to sound high-quality enamel and dentine margins. The pulpal wall remains unprepared at this stage. Note, evidence of an underlying crack within the tooth, running from mesial to distal, begins to become apparent. This is a typical finding in long-standing carious lesions, secondary to a loss of structural integrity

Enamel undermined and demineralised by the carious process was removed using a diamond fissure bur (Kerr, Bioggio, Switzerland) in a turbine dental handpiece (W&H, Bürmoos, Austria) under copious water coolant to develop access form, thereby revealing the extent of the underlying carious dentine (Fig. 3). Subsequently, clearing of the peripheral amelodentinal junction was conducted using a large steel rosehead bur (Prima Dental Group, Gloucester, England) at slow speed under copious water coolant (Figures 4a, 4b and 4c). The periphery of the carious lesion should be cleared to sound dentine using a sequence of sterile rosehead burs from largest to smallest. The largest rosehead bur that can fit within a cavity is recommended initially to clear soft dentine, as this drill sequence prevents gouging into the dentine and resultant iatrogenic tooth structure removal. To permit the insertion of a matrix band, the mesial proximal contact was broken (Figures 5a, 5b and 5c). Unsupported enamel was also removed as it is prone to fracture under functional loading once restored.^11^ The function of creating a periphery of sound hard dentine, amelodentinal junction and sound enamel is to create a peripheral seal when the adhesive restoration is placed. The creation of the peripheral seal zone enables the adhesive bond to be preserved for the long-term.^18^ In such circumstances, dentine bonding should be similar to that carried out in the healthy tooth. Thus, if the tensile strength of a resin bond to the amelodentine junction is 51.5 MPa, bonding to dentine should mimic this value.^18^ Bonding to the peripheral seal zone generates a bond strength of 45-55 MPa according to the literature.^18^ Enamel bevelling was not conducted in this clinical technique. Enamel bevelling is generally not recommended in posterior teeth since bevelled margins are more difficult to detect and the resin composite layer is more prone to marginal staining and paramarginal fractures under long-term occlusal loading.^19^^,^^20^

**Fig. 6:**
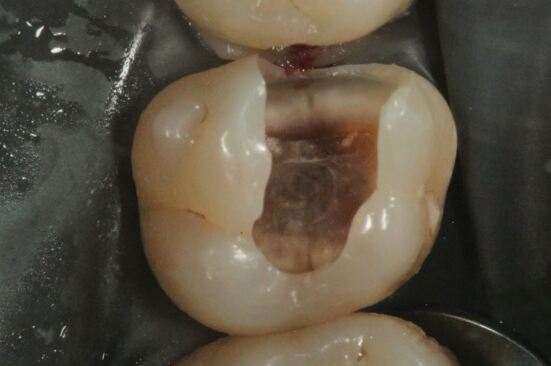
In the final steps of tooth preparation, caries that lies over the pulpal wall is excavated by hand, with the final endpoint of caries excavation being determined by tactile sensation rather than by the colour of the so-called caries-affected dentine

Hand excavation was conducted to remove carious tissue on the pulpal walls (Fig. 6). The final endpoint of excavation of carious tissue should be determined by the texture of the lesion, rather than the colour.^11^ Removing carious tissue using hand excavation enables the operator to have tactile sensation. Using rotary instruments considerably reduces tactile feedback during selective excavation and can risk iatrogenic removal of excessive tooth structure at this key site. In this particular case, following completion of selective caries excavation, a 'crack' running from mesial to distal on the 26 was noted at the base of the cavity (Figures 5b, 5c, 6 and 8b). The implications of this will be discussed under the heading 'Long-term prognosis'.

Although it is recommended to place a hydraulic calcium silicate or a conventional glass ionomer cement on the dentine barrier before placing a definitive restoration, no underlying layer of pulp protection materials were placed in this case example as it still remains elusive whether the presence of an underlying substrate will compromise the strength and longevity of the overlying methacrylate resin-based composite restoration.^15^^,^^21^ The literature also provides no evidence supporting any auxiliary clinical benefit in placing indirect pulp protection to avoid post-operative sensitivity.^2^^,^^4^^,^^22^ In this case, since the carious lesion was moderate to deep and not deep or extremely deep, a decision was made that a liner was not essential, as the probability of a micro-exposure was low.

One of the 'golden triangles' of minimally invasive dentistry is the understanding of the chemistry and handling of adhesive materials used to restore a cavity.^6^ In this case, a fifth-generation bonding system was used: a two-step etch-and-rinse system.

**Fig. 7:**
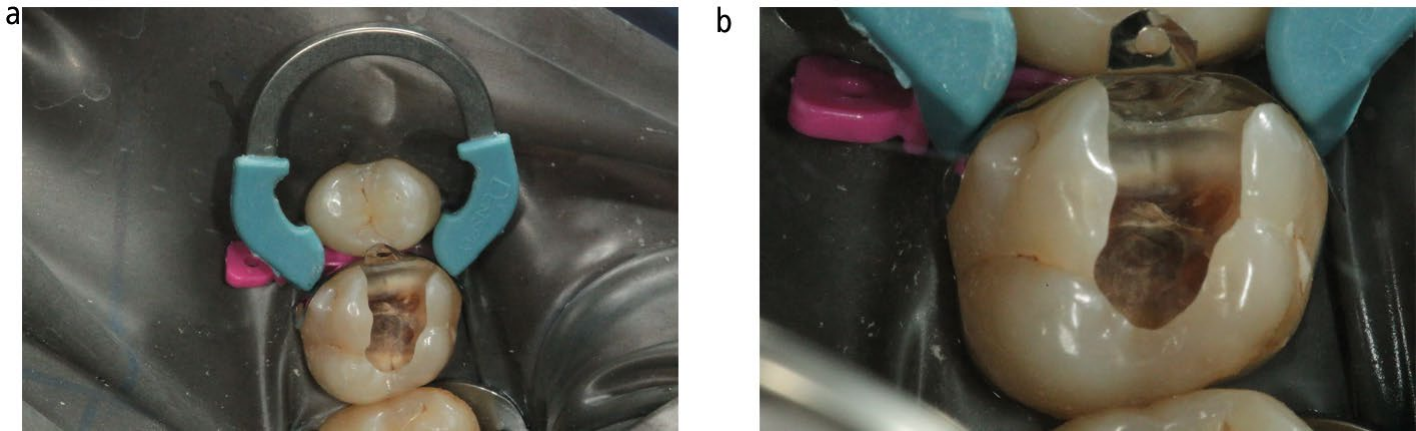
a, b) The use of and correct positioning of an anatomical matrix system offers many benefits for contemporary adhesive restorative materials. The superior contouring of proximal contacts that can be achieved with such systems creates more structural integrity in the resultant restoration and also prevents food impaction

An anatomical V-ring sectional matrix system (Palodent V3, Dentsply Sirona, North Carolina, USA) was placed on the tooth and a wedge was placed in between the 25 and 26 to ensure its tight adaptation to the 26 (Figures 7a and 7b). The pre-curved V-ring sectional matrices have been shown to be advantageous over circumferential matrix systems in providing properly contoured proximal contacts, which will thereby render the marginal ridge less susceptible to chipping and fracture.^4^^,^^23^ Moreover, it will recreate embrasure anatomy and 'tight' contacts that will facilitate biofilm removal proximally and reduce food impaction respectively.^11^

## Bonding protocol

**Fig. 8:**
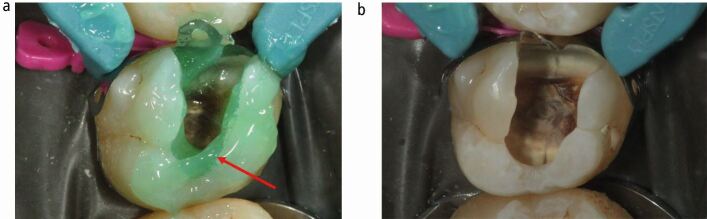
a, b) The total-etch technique recognises that enamel and dentine are fundamentally two different substrates which benefit from a nuanced approach to their bonding. The inclusion of air bubbles demonstrates that the etch has been agitated on the tooth surface

Enamel was etched with 37% orthophosphoric acid for 30 seconds first, followed by etching of dentine for 15 seconds. The surface of unetched enamel is smooth and has little potential for bonding by micro-mechanical retention. Acid etching enamel modifies the surface of enamel by demineralisation of the hydroxyapatite crystal, creating micro-porosities that enable adhesive resins to flow by capillary action forces into them, allowing for micro-mechanical retention of the methacylate resin-based composite. Acid etching of dentine causes demineralisation of intertubular and peritubular dentine.^24^ Etching enamel for a longer duration than dentine enables sufficient surface roughness to be created in order to yield increased bond strength when the resin adhesive is applied on the surface.^8^^,^^25^^,^^26^ The frosted appearance of enamel after etching allows the clinician to visualise the extent and efficacy of the etch (Figures 8a and 8b).^27^ The total etch technique on dentine removes the smear layer, thereby exposing the dentinal tubules.^8^^,^^24^ Bonding to dentine similarly utilises a micromechanical bond. This mechanical bond originates from the network of interlocking monomers with the collagen fibrils and the formation of resin tags from the adhesive diffusing into the demineralised enamel and dentine structure.^8^^,^^24^ The combination of primer and adhesive essentially creates a critical 'hybrid layer' on the collagen matrix which forms the foundation to a successful adhesive bonding of overlying composite resin. Over-etching dentine longer than the recommended time will result in decreased longevity of dentine bond strength, as the adhesive may not be able to infiltrate the demineralised collagen matrix in order to create this hybrid layer.^28^^,^^29^

After application of the acid etch and thorough rinsing with water, the next step consists of applying the primer and adhesive. In fifth-generation dentine bonding systems, these two immiscible liquids are both contained within one bottle. It is important that the bottle is well-shaken before use to ensure adequate primer and adhesive dispersion. The bonding agent should not be applied to desiccated dentine as some moisture maintains the spaces between the collagen fibrils, thereby preventing the collapse of the collagen fibril network and encouraging hydrophilic resin primer infiltration into the dentinal tubules.^24^^,^^30^ A doubled-layer application of the bonding agent on dentine was used, as this technique has been shown to provide a higher bond strength and durability.^31^ The first layer will not have the right ratio applied on the cavity preparation but will improve 'wettability' for the subsequent layer of resin adhesive.

Evaporation of the solvent in the bonding agent is a necessary step before curing. Although the solvent is an important carrying medium incorporated in the formulation of the bonding agent to allow infiltration of resin into the moist dentine collagen matrix, it is important to evaporate the solvent (acetone) with dry, uncontaminated compressed air before light curing.^24^ This essential step facilitates the polymerisation reaction of the resin adhesive and prevents a porous structure of the cured adhesive within the adhesive-dentine interface.^32^ Primer and adhesive was light cured for 40 seconds, with the light curing unit placed as close to the resin composite as possible.

**Fig. 9:**
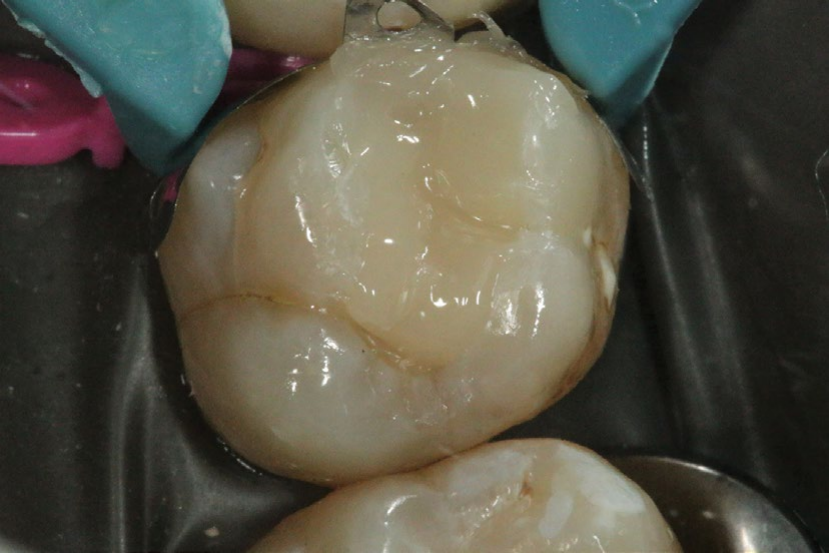
Methacrylate resin-based composite is incrementally cured in three layers of maximum 2 mm thickness. Such a placement strategy ensures complete light polymerisation of the restorative material whilst controlling for stresses which develop at the resin-tooth structure interface

**Fig. 10:**
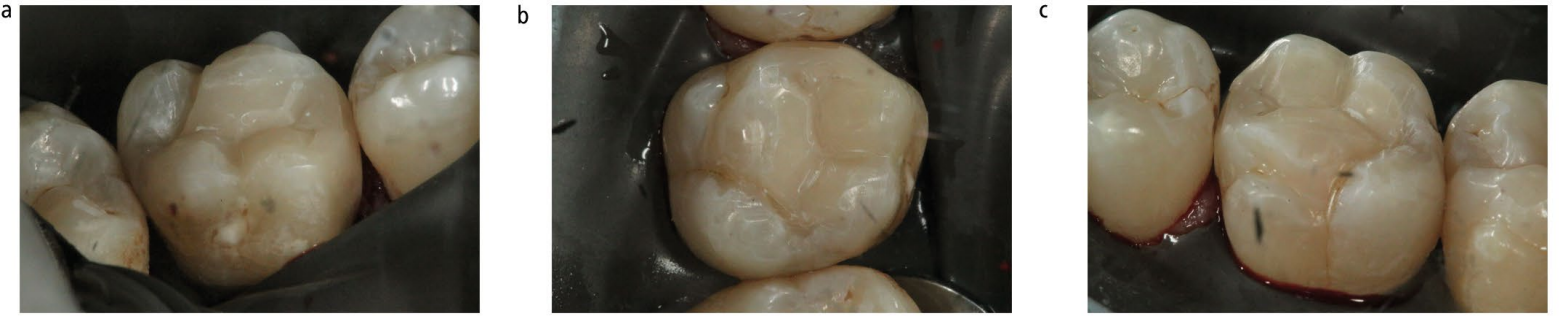
a, b, c) Prior to removal of the rubber dam, the restoration is shaped and polished with intensive finishing diamonds and interproximal finishing strips. The restoration must conform to the existing occlusal scheme, which should be confirmed with articulating paper following removal of the rubber dam

When methacrylate resin-based composite is cured, polymerisation shrinkage occurs and imparts residual stresses on the tooth. According to the literature, residual shrinkage stresses can lead to inadequate adaptation of composite to the cavity, microcrack propagation and loss of marginal seal, with associated post-operative sensitivity and microleakage.^33^ Therefore, consideration of the C-factor is important when restoring a cavity. C-factor is the ratio between a bonded and unbonded surface.^33^ Higher C-factor leads to an increased risk of debonding at the resin-dentine interface, with resultant microleakage.^33^^,^^34^^,^^35^ This cavity was filled with three horizontal increments in order to reduce the C-factor and achieve adequate bond to the cavity floor (Fig. 9).^35^ The final restoration was polished using intensive finishing diamond burs (Meisinger, Neuss, Germany) and proximal areas were polished with interproximal strips (3M, Minnesota, USA) to remove resin flash (Fig. 10). Optimum occlusal relationship was confirmed using articulating paper.

With advances in resin composite technology, bulk-fill flowable composites have become readily available which has thereby led to alternative incremental restorative techniques. The rationale behind the bulk-fill incremental technique aims to restore the majority of the cavity with a less-viscous methacrylate resin-based composite of 4 mm in thickness, followed by a 2 mm capping of high-viscosity methacrylate resin-based composite. This technique aims to reduce resin composite placement time, making it more time-efficient compared with the conventional oblique incremental technique. Moreover, the 4 mm bulk-fill technique with flowable resin composite has been demonstrated to have comparable polymerisation shrinkage stress and clinical effectiveness compared to the conventional layering technique.^36^^,^^37^^,^^38^ However, the polymerisation shrinkage of bulk-fill resin composites is dependent on several factors, such as filler content, polymerisation kinetics and degree of conversion.^36^ In addition to the insufficient clinical data available regarding the shrinkage behaviour of these composite types, their behaviour and formulation characteristics will differ among the various product manufacturers.^36^^,^^39^^,^^40^

Glass ionomer cements were first developed in the 1960s but continue to be used as restorative materials to this day. Their adhesion to tooth structure coupled with their supposedly cariostatic properties due to fluoride release continues to make glass ionomer cements relevant in clinical practice, particularly when restoring primary teeth via the atraumatic restorative technique.^41^ However, the evidence behind the fluoride releasing capabilities and ability to reduce the incidence of secondary caries of glass ionomers remain questionable.^42^^,^^43^ Glass ionomers have also yet to be advocated for posterior dentitions due to their inferior tensile strength in load-bearing sites.^42^^,^^44^ Despite attempts to reinforce the glass ionomer matrix with the addition of filler types, these strategies are still unable to produce mechanical properties similar to resin-based composites.^45^

## Long-term prognosis

**Fig. 11:**
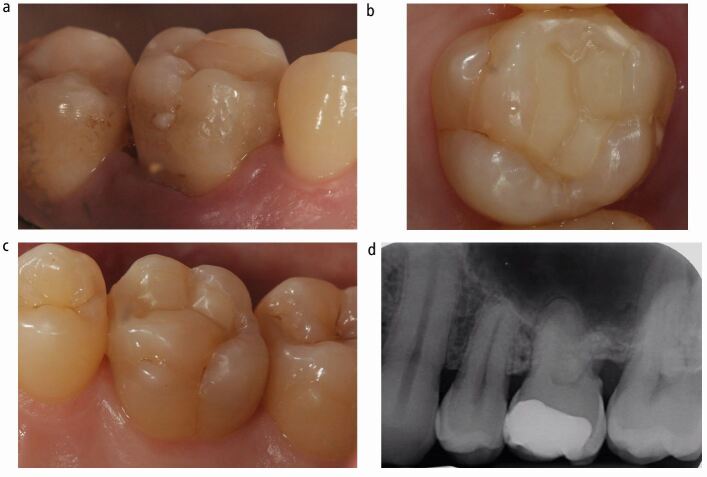
a, b, c, d) At 12-month post-operative review, the restorative margins should be examined and be clinically intact. Pulpal sensibility should be confirmed with electrical and cold testing whilst percussive sensitivity should be excluded. An updated intraoral periapical radiograph or bitewing radiograph may be made to permit future restorative planning for the tooth or teeth in question

When managing deep carious lesions, review of the final restoration is part of holistic patient care to eliminate the failure of the tooth-restorative complex. Clinicians should examine surface irregularities and the marginal integrity of the restoration, ensuring it does not become a plaque stagnation area.^8^^,^^46^ Selectively excavating caries and leaving some carious tissue behind beneath a restoration poses a potential dento-legal concern. The rationale behind the technique should therefore be communicated to the patient with an emphasis on recall to confirm pulpal health of the tooth over time.^15^ In this case, the 26 tooth was re-assessed at 12 months (Fig. 11). Assessment involves visual and tactile clinical examination, percussion testing, cold and electrical pulpal sensibility testing and intraoral radiography. Pulpal vitality and apical health was maintained in this case example.

As highlighted previously, in this particular case example, a mesial-distal 'crack' was detected clinically at the base of the 26 cavity preparation. This is likely due to a reduction in structural integrity of this tooth, subsequent to the loss of its mesial and oblique marginal ridges caused by the unchecked progression of the proximal carious lesion, resulting in an overall reduction of tooth stiffness.^47^ The direct composite restoration is a conservative, time-efficient and cost-effective temporary solution which also 'splints' the tooth in the short-term and provides a seal in 'cracks' which would otherwise be colonised by bacterial biofilm.^48^ However, without cuspal coverage, repeated cyclical fatigue loading on the restoration, which would occur through typical function, is likely to compromise the integrity of the adhesive layer and consequently compromise the splinting effects of the restoration.^49^ Hence, cuspal coverage will likely be mandated in the future, providing that periodontal and carious process stability can been achieved. The combination of periodontal disease and root shortening in this example renders the prognosis of the 26 tooth to be guarded. As such, a joint decision should be made in conjunction with the patient regarding the cost-benefit of proceeding with indirect cuspal coverage restoration in a case such as this. Should an indirect cuspal coverage restoration be undertaken, the methacrylate resin-based composite restoration is likely to serve as a satisfactory definitive core material for this purpose, even with a selective approach to carious tissue removal.

## Conclusion

This case report underpins the advantages of conservative management of carious lesions in its operative context for moderate-to-deep depth carious lesions. As the famous adage of cariologist Edwina Kidd states, 'the seal is the deal'. So long as the cavity has an adequate hermetic seal, selectively excavating and leaving carious tissue behind should not be of concern and will neither result in clinically significant pulpal inflammation, nor progression of the carious lesion. Although operative management of carious lesions is important, non-operative management, such as diet modification through the limitation of fermentable carbohydrates, plaque control and fluoride exposure, is even more critical in controlling the carious process within the oral environment.^50^
